# Cardiac Myxoma among Patients Undergoing Cardiac Surgery in a Tertiary Care Center: A Descriptive Cross-sectional Study

**DOI:** 10.31729/jnma.6538

**Published:** 2022-02-28

**Authors:** Prabhat Khakural, Ravi Baral, Anil Bhattarai, Bhagawan Koirala

**Affiliations:** 1 Department of Cardiothoracic and Vascular Surgery, Manmohan Cardiothoracic Vascular and Transplant Center, Maharajgunj Medical Campus, Institute of Medicine, Maharajgunj, Kathmandu, Nepal

**Keywords:** *embolism*, *heart neoplasms*, *myxoma*

## Abstract

**Introduction:**

Heart neoplasms are rare tumors. Myxoma is the commonest primary benign tumor of the heart presenting with features of obstruction, arrhythmia, and embolism. Surgical excision of the tumor is the gold standard of treatment. The aim of the study is to find out the prevalence of cardiac myxoma among all cardiac surgeries operated during the study period.

**Methods:**

A descriptive cross-sectional study was done among 3800 patients undergoing surgery for cardiac tumors in a tertiary care center after obtaining approval from the Institutional Review Committee (Reference number- 36/(6-11)E2/077/078). The data was collected retrospectively from August 2012 to August 2020 using convenience sampling method. Statistical analysis was performed using Microsoft Excel 2016. Point estimate at 95% Confidence Interval was calculated along with frequency, percentage, mean and standard deviation.

**Results:**

There were 26 (0.68%) (0.42-0.94 at 95% Confidence Interval) myxoma among 3800 cardiac surgeries performed over eight years. The mean age of the patients was 54.76±14.31 (range 17-75) years. Twenty (76.92%) patients were females. The commonest presenting symptom was shortness of breath in 19 (73.07%) patients. En masse excision with the closure of the atrial septal defect was the principal surgical technique. The mean Intensive Care Unit stay and hospital stays were 2.92±1.29 and 6.26±2.61 days respectively. There was no perioperative mortality.

**Conclusions:**

Cardiac myxoma was the most common cardiac tumor encountered as in other studies.

## INTRODUCTION

Cardiac tumors are rare tumors with a prevalence of 0.0017 to 0.28%.^1^ Metastatic tumor of the heart is commoner than benign tumors with a prevalence of 1% and 0.1%, respectively.^2^ The ratio of occurrence of primary benign to malignant cardiac tumors is 2:1.^3^ Myxoma and sarcoma are the commonest benign and malignant cardiac tumors respectively.^4^

Myxoma affects women in the 3^rd^-6^th^ decades of life more frequently.^5^ Such patients present with features of valvular obstruction, heart failure, arrhythmia, embolization, and constitutional symptoms.^6^ Heart neoplasms are diagnosed by echocardiogram, computed tomography, magnetic resonance imaging.^7^ Benign tumors are treated with complete surgical resection whereas malignant tumors need to be completely excised as far as possible and treated with adjuvant chemotherapy and radiotherapy.^8^

Researches on the cardiac tumor from Nepal are scarce. This study is intended to find out the prevalence of cardiac myxoma among all cardiac tumors operated in our center during the study period.

## METHODS

This was a descriptive cross-sectional study conducted in the Department of Cardiothoracic Vascular Surgery in Manmohan Cardiothoracic Vascular and Transplant Center, Maharajgunj, Kathmandu, Nepal. The data was collected retrospectively from August 2012 to August 2020 through medical records. Ethical approval was obtained from the Institutional Review Committee (IRC) of Institute of Medicine (Reference number: 36/ (6-11)E^2^/077/078). Convenience sampling technique was used and sample size was calculated according to the formula:

n = Z^2^ × p × q / e^2^

  = (1.96)^2^ × 0.5 × (1-0.5) / (0.02)^2^

  = 2401

Where,

n = minimum required sample size
Z = 1.96 at 95% of Confidence Interval (CI)
p = prevalence taken as 50 % for maximum sample size
q = 1-p
e = margin of error, 2%

Adding a 10% non-response rate, the calculated sample size was 2641. However, 3800 cases were taken.

Data collected from patient files included patient characteristics-age, gender, New York Heart Association (NYHA) class, presentation of cardiac tumors, intraoperative tumor size, cardiopulmonary bypass time, aortic cross-clamp time, postoperative hours of ventilation, mediastinal drainage, postoperative complications, duration of hospital and Intensive Care Unit (ICU) stay, histopathology of a tumor, in-hospital and long-term survival. Long-term survival was studied by reviewing the follow-up record and telephonic interview.

Data were recorded and descriptive statistics like frequency, percentage, mean and standard deviaton were calculated using Microsoft Excel 2016. Point estimate at 95% Confidence Interval was also calculated.

## RESULTS

Among 3800 cardiac surgeries, 26 (0.68%) (0.42-0.94 at 95% Confidence Interval) were performed for cardiac myxoma. The mean age of the patients with myxoma was 54.76±14.31 years (range 17-75). Eleven (42.30%) of the patients were in the 50-59 years group. The majority of the patients were females, with a female to male ratio of 3.3:1. The mean duration of symptoms was 119.84±134.11 days. Two cases were diagnosed incidentally while being worked up for hypertension. The presenting symptoms were shortness of breath, palpitation, cough, anorexia with weight loss, and features of embolism (Table 1). Only six (23.07%) patients with cardiac myxoma had a history of smoking. The most common tumor location was the left atrium followed by the right atrium. Tricuspid regurgitation was the commonest associated valvular dysfunction followed by mitral regurgitation (Table 1). One patient had functional mitral stenosis whereas another one had an organic stenotic lesion.

**Table 1:** Pre-operative variables (n = 26).

Variables		n (%)
**Gender**	Male	6 (23.07)
	Female	20 (76.92)
**Presenting symptoms**	Dyspnoea	19 (73.07)
	Palpitation	14 (53.84)
	Embolism	4 (15.38)
	Fever	1 (3.84)
	Anorexia and weight loss	4 (15.38)
	Cough	4 (15.38)
**NYHA class**	I	1 (3.84)
	II	16 (61.53)
	III	9 (34.61)
	IV	-
**Smokers**		6 (23.07)
**Echocardiogram findings**	Tumor site	
	Left atrium	23 (88.46)
	Right atrium	2 (7.69)
	Left ventricle	1 (3.84)
	Tumor protrusion through mitral valve	14 (53.84)
	Mitral regurgitation	6 (23.07)
	Tricuspid regurgitation	8 (30.76)
	Severe pulmonary artery hypertension	3 (11.53)

All the patients with myxoma underwent complete excision of the tumor. A separate right and left atriotomy (Bicameral approach) was the most commonly employed approach (Table 2). A total of 14 (53.84%) patients had myxoma with a broad base whereas the remaining tumors were pedunculated. Piecemeal excision of the very friable tumors was performed. The majority of the tumors were excised with a wide rim of interatrial septal attachment followed by the repair of the interatrial septum either with a pericardial patch or direct closure (Table 2). Aortic cross clamp time was 30.46±14.52 minutes and cardiopulmonary bypass time was 46.19±18.19 minutes. Adjunct procedures performed included tricuspid valve repair, mitral valve repair, mitral valve replacement, and coronary artery bypass grafting.

**Table 2:** Intra-operative variables (n = 26)

Variables		n (%)
**Approach**	Bicameral (left atriotomy and right atriotomy)	22 (84.61)
	Left ventriculotomy	1 (3.84)
	Right atriotomy	3 (11.53)
**Tumor attachment**	Pedunculated	12 (46.15)
	Sessile	14 (53.84)
**Tumor excision**	En masse	21 (80.79)
	Piecemeal	5 (19.23)
**Procedure for tumor**	Excision with suturing of endocardium	7 (26.92)
	Excision with IAS rim with direct closure of defect	6 (23.07)
	Excision with IAS rim with pericardial patch closure of defect	13 (50.00)
**Adjunct procedure**	Kay's annuloplasty of TV	1 (3.84)
	Ring annuloplasty of TV	1 (3.84)
	Commissural advancement of mitral valve	2 (7.69)
	Mitral valve replacement	1 (3.84)
	Coronary artery bypass grafting	1 (3.84)

The majority of the patients were extubated the same day, had insignificant mediastinal drainage and stayed in ICU and hospital for an average period of 2.92±1.29 and 6.26±2.61 days respectively (Table 3). Seven (26.92%) of the patients had arrhythmias requiring intervention (Table 4). One patient developed hemiparesis in the postoperative period which got improved by the time of discharge. The CT head of the patient did not reveal ischemia or hemorrhage. There was no perioperative mortality.

**Table 3:** Post-operative variable.

Variables	Value (Mean±SD)
Ventilation support (hours)	7.91±6.27
Mediastinal bleeding (ml)	272.50±192.66
ICU stay (days)	2.92±1.29
Hospital stay (days)	6.26±2.61
Drain placement (days)	1.57±0.64

**Table 4:** Post-operative complications.

Variables	n (%)
Arrhythmia	7 (26.92)
Cerebrovascular accident	1 (3.84)
Heart failure	1 (3.84)
Renal impairment requiring dialysis	1 (3.84)
Perioperative mortality	-

The patients followed up for a mean period of 1.49 years (range 7 days to 6.5 years). A review of medical records and telephonic interviews identified five late deaths.

## DISCUSSION

In our study 89% of surgically treated cardiac tumors was myxoma similar to Keeling IM, et al. who found that 86% of surgically treated cardiac tumors were cardiac myxomas.^9^ The incidence of cardiac myxoma is 0.3% in patients undergoing on-pump cardiac surgeries.^10^ Cardiac myxoma excision contributed to 0.68% of cardiac surgeries in our centre. Of the primary cardiac tumors, cardiac myxoma were 92.85% and 7.14% were malignant tumors. The majority of the patients, 20 (76.92%) with myxoma were females and the mean age was found to be 54.76±14.31 years. In a study by Mandal sc, et al. the female to male ratio of myxoma occurrence ratio was 1.9:1 and (43%) of patients were in the 4^th^ to 5^th^ decade of life.^11^ The mean age of the patients was 55 years (range 22-79 years) in a study by Keeling IM, et al.^9^ The mean duration of onset of symptoms to diagnosis was 119.84±134.11 in our population.

The most common presenting complaint of the patients included shortness of breath followed by palpitation and 16 (61.52%) patients were in NYHA class II, in our study which is similar to that reported in other studies. Fifty-one percent 51.0% presented with dyspnea and 65.78% were in NYHA Class II.^10,12^ Two (7.69%) patients had a preoperative stroke and two (7.69%) had femoral artery embolism in our center. In another study from Pakistan, 50% of patients had neurological symptoms at presentation including stroke and transient ischemic attack, and 14% had limb ischemia secondary to embolism.^13^ Similarly preoperative atrial fibrillation was seen in 3.84% in our study. Cianciulli TF, et al. found 9.3% patients had atrial fibrillation.^5^ Similar to our findings, 82.4%-86.6% of the myxoma was located in the left atrium attached to the interatrial septum.^11,14^ Protrusion of the tumor through the mitral/ tricuspid valve can give rise to symptoms of valvular obstruction, increased pulmonary artery pressure, and tricuspid regurgitation. Fourteen (53.84%) of our patients had myxoma protrusion through the mitral valve whereas eight (30.76%) patients had varying grades of tricuspid insufficiency.

Various operative approaches to myxoma excision have been described in literature like minimally invasive, endoscopic, and robotically-assisted techniques, however, median sternotomy remains the most commonly performed one.^2^ All our patients underwent median sternotomy. The tumor size ranged from 2cm to 8cm, with a mean size of 4.13±1.39cm. The majority of the tumors 53.84% were broad-based. Tumor size ranged from 1cm to 12cm and 67.6% to 85.48% of the tumors were pedunculated in other studies.^14,15^ Complete excision of the tumor is of utmost importance in managing cardiac tumors. Hence the commonest approach for left atrial myxoma excision was the bicameral approach in 22 (84.61%) patients. 21 (80.79%) of our patients underwent en masse excision of the tumor and 13 (50%) underwent excision of the tumor along with a portion of the interatrial septum and the defect closure with an autologous pericardial patch. Similar approaches and techniques were followed by other surgeons.^16^ Various studies have shown patients with myxoma excision may need concomitant procedures like mitral valve and tricuspid valve repair or replacements, coronary artery bypass grafting.^16^ Tricuspid repair was done by Kay's annuloplasty in one patient and ring annuloplasty in another patient. One patient underwent mitral valve replacement with excision of the tumor and another patient underwent coronary artery bypass grafting to a distal right coronary artery. The mean cardiopulmonary bypass time and aortic cross-clamp time were found to be 46.19±18.19 minutes and 30.46±14.52 minutes in our study. However longer mean cardiopulmonary bypass time (80.7±39.0 minutes) and mean aortic cross-clamping time (51.3±27.5 minutes) have also been reported.^17^

The postoperative duration of ventilation was 7.91±6.27 hours in our patients. The mean mediastinal drainage was 272.50±192.66ml and the drains were placed for a mean duration of 1.57±0.64 days. However, packed cells transfusion was required in two patients only. One patient had hemiparesis; seven patients had arrhythmia in the post-operative period. Two patients had supraventricular tachycardia, two had atrial fibrillation with fast ventricular rate, managed by chemical cardioversion and two patients had ventricular fibrillation requiring electrical cardioversion. One patient had a run of ventricular tachycardia abolished by Lidocaine. One patient with postoperative cardiac failure with renal impairment responded well to medical management and three sessions of dialysis. None of the patients had re-exploration for bleeding or tamponade, sepsis, and surgical site infection.

The mean postoperative intensive care unit stay and hospital stays were 2.92±1.29 days and 6.26±2.61 days respectively. In the hospital, perioperative mortality was not seen. Most of our patients were from remote areas and hence most of them did not follow up for a long period. The mean follow-up period was 1.49 years. Long-term survival was seen in 21 (80.76%) patients. There were two late in-hospital mortalities. One patient presented to the Emergency room with acute right lower limb ischemia of 24 hours duration and cardiac failure, one year after myxoma excision. The echocardiographic evaluation revealed recurrence of left atrial myxomatous tumor approximately 2cm in size. However, the patient crashed in an emergency and succumbed to the disease before she could be shifted to the operating theatre. Another patient had expired following tricuspid valve replacement for severe tricuspid regurgitation, three years after right atrial myxoma excision. In a telephonic interview, it was found that three patients (above 72 years of age) died at home.

In a study from India, 7.89% of patients had supraventricular arrhythmias, 5.26% of patients had an atrioventricular block requiring temporary pacing and the early death rate was 5.88%. 15.78% of patients were lost to follow-up. There were no late deaths and recurrence.^12^ In a Greek study of 153 patients, the mean ICU stay and hospital stay were 2.0±0.9 days and 8.02±2.8 days respectively. Postoperatively, atrial fibrillation was seen in 5.9%, permanent pacemaker insertion was required in 5.2% and in-hospital mortality due to sepsis was 0.7%. Recurrence was found in 3.3% of patients in a mean follow-up of 3.7±4.3 years.^14^

The limitations of the study were that the small sample size and it was a single-center study.

## CONCLUSIONS

Cardiac myxoma was the most common cardiac tumor in our study as in other published studies performed. Proper diagnosis and management of cardiac myxoma could result in complete and safe excision with low perioperative morbidity and mortality.
